# Liver Metastases in Prostate Carcinoma Represent a Relatively Aggressive Subtype Refractory to Hormonal Therapy and Short-Duration Response to Docetaxel Monotherapy

**DOI:** 10.14740/wjon903w

**Published:** 2015-02-14

**Authors:** Arsh Singh, Naga K. S. Cheedella, Shams A. Shakil, Frederick Gulmi, Dong-Sung Kim, Jen C. Wang

**Affiliations:** a Division of Hematology/Oncology, Brookdale University Hospital Medical Center, Brooklyn, NY, USA; b Department of Urology, Brookdale University Hospital Medical Center, Brooklyn, NY, USA; c Department of Pathology, Brookdale University Hospital Medical Center, Brooklyn, NY, USA

**Keywords:** Hepatic metastasis, Prostate carcinoma

## Abstract

**Background:**

We report a single institution’s experience from a small series of patients suggesting that liver metastasis in metastatic castration-refractory prostate cancer (mCRPC) represents a relatively aggressive subtype that is refractory to hormonal manipulation treatment, including luteinizing hormone-releasing hormone agonist (LA) and abiraterone (Ab) therapy, although docetaxel is briefly effective.

**Methods:**

Between 2007 and 2013, six patients with prostate cancer with liver metastases were analyzed. Biochemical response was defined as > 50% decrease in prostate-specific antigen (PSA) value.

**Results:**

Two patients who presented with liver metastases died in less than 3 months after LA therapy. Two out of three patients (one died while receiving chemotherapy) received Ab after chemotherapy did not show any response and died while on therapy. One patient who presented with lung metastases initially received LA therapy and progressed on it with liver metastases in < 6 months. Thus, five of six patients did not respond to hormone therapy including LA and Ab. Three patients who received docetaxel after LA therapy had more than 50% objective PSA response with a mean survival of 4 months.

**Conclusions:**

No literature addresses the response to hormone treatment in hepatic metastasis in prostate carcinoma. This small series suggests that liver metastases in prostate carcinoma represent a relatively aggressive subset against which hormonal therapy, including the LA and Ab, appears to be ineffective. Although our patients responded to docetaxel chemotherapy, their responses were of short duration. A further clinical trial involving more patients will be necessary to substantiate our findings.

## Introduction

Prostate cancer is the second most common cause of cancer death in men both in the United States and Europe. Approximately one in six men in the United States will develop prostate cancer. Roughly 30% of all newly diagnosed cancers in males are prostate cancer [1]. The estimated number of new cases of prostate cancer in the United States for 2013 is 238,590, and the estimated number of resulting deaths is 29,720 [1]. The high incidence/mortality ratio (8:1) suggests that the disease is lethal for only some men. Autopsy series have shown prostate cancer prevalence from 16.1% to 33.3% [2, 3]. Also, not all patients who harbor prostate cancer develop clinical disease. Histological evidence of prostate cancer has been shown through autopsy studies to be present in 30% of men above the age of 50 [4]. However, only 9% of these men actually develop clinical disease [4]. These facts not only highlight a variable clinical course of prostate cancer but also reflect a broad spectrum of tumor biology and its behavior.

Prostate cancer requires a risk-adapted approach in which therapeutic options are distinct, depending on clinical states from pre-diagnosis to death. Our understanding of the disease shows that, in some patients, the risk of death from non-cancer-related causes exceeds that of prostate cancer while in others, especially symptomatic metastatic castration-resistant prostate carcinoma (mCRPC), a more aggressive and immediate intervention is required. This speaks to the biology of this disease, which for the most part remains elusive. Here, we report a single institution’s experience from a small series of patients suggesting that liver metastasis in mCRPC represents a relatively aggressive subtype that is refractory to hormonal manipulation treatment, including luteinizing hormone-releasing hormone agonist (LA) and abiraterone (Ab) therapy, although docetaxel is briefly effective.

## Patients and Method

Data were retrospectively collected from our institution’s medical records department specifically for patients with metastatic prostate cancer from 2007 to 2013. Six patients who developed liver metastasis during the course of their disease were identified. Liver metastasis was histologically confirmed in five out of six patients, while one was confirmed radiologically. Charts were thoroughly reviewed, and patients were followed from the time of diagnosis of liver metastasis until the preparation of this manuscript. Patients’ characteristics collected were age, date of original prostate cancer diagnosis, Gleason score, performance status, prostate-specific antigen (PSA) value and its trend, time until progression, time until liver metastasis, treatment given, response to treatment, number of cycles of chemotherapy if given, histological and radiological features of metastasis, ALP, ALT/AST, Hb, and survival after liver metastasis development with or without treatment. Survival was defined as the time from the date of diagnosis of liver metastasis until the date of death or last follow-up. PSA response was defined as at least a 50% decline in PSA value after the start of treatment. All radiological images were viewed and reviewed with a certified radiologist. Histological and immunohistochemical studies of liver biopsies were stained with neuroendocrine marker to exclude neuroendocrine tumor. The polyclonal antibodies used were mainly CD56, chromogranin-A, synaptophysin, thyroid transcription factor (TTF-1), PSMA, PSA, CAM5.2, CDX2, CEA, CK7, and CK20.

## Results

In the data collected retrospectively at our institution from 2007 to 2013, we identified six patients with prostate cancer who developed liver metastasis at some point during their disease course. The clinical characteristics of these six patients are shown in Table 1. Median age at the time of liver metastasis was 68.5 years. The ECOG performance status was generally good. Gleason score was at least 8 in four out of six patients (two unknown) and median PSA was 149, suggesting an aggressive disease from the onset. Although median PSA value at diagnosis of prostate cancer was 149, the median PSA at the time of liver metastasis was lower at 100.5, likely reflecting the effect of hormonal therapy. At the time of diagnosis of prostate cancer, two patients did not have metastatic disease; among the four who presented with metastatic disease, two had metastasis to bone while the third patient had metastasis to bone and liver. The fourth patient was diagnosed with metastasis to liver, bone, and lung. The median time to development of liver metastasis was 15.5 months.

**Table 1:** Patient Characteristics Prior to and at the Time of Diagnosis of Liver Metastasis

Characteristic	N
Median age	68.5 (59 - 81)
ECOG performance status	1 (3), 2 (3)
Gleason score	8 (2), 9 (2), unknown (2)
Median PSA at diagnosis of prostate cancer	149
Chemotherapy given prior to liver metastasis	None
Median time to development of liver metastasis	15.5 months
PSA at the time of liver metastasis	100.5
Hemoglobin	9.95 (7.4 - 14)
Alkaline phosphatase	233.5 (59 - 524)
AST	72 (24 - 170)
ALT	46.5 (12 - 79)
Albumin	3.8 (3.2 - 4.2)
Metastasis to other sites	Lung (2), bone (3), none (3)

In the five patients with histological confirmation of liver metastasis, we ruled out neuroendocrine differentiation of the prostate cancer at the metastatic site after reviewing liver biopsies with the pathologist for histological and immunohistochemical studies. The five specimens did not show any neuroendocrine or small cell carcinoma differentiation (Table 2). All adenocarcinoma were positive for either PSA and/or PSMA on IHC while none expressed chromogranin-A, synaptophysin, or CD56 (N-CAM) except one focally positive for synaptophysin. Focal positivity for synaptophysin was seen in very few cells and was considered nonspecific [5].

**Table 2:** Pathological Analysis of Liver Metastasis

IHC	Patient 1	Patient 2	Patient 3	Patient 4	Patient 5	Patient 6
Chromogranin-A	Negative	Negative	Negative	Negative	N/A	Negative
Synaptophysin	Negative	Negative	Negative	Negative	N/A	Focally positive
CD56	Negative	Negative	Negative	Negative	N/A	Negative
CK7	Negative	Negative	Negative	Negative	N/A	N/A
CK20	Negative	Negative	Rare positive	Negative	N/A	N/A
TTF-1	Negative	Negative	Negative	Negative	N/A	N/A
PSMA	Positive	Negative	Positive	Positive	N/A	Positive
PSA	Negative	Positive	Negative	Negative	N/A	Positive
Final histology	Adeno CA	Adeno CA	Adeno CA	Adeno CA	N/A	Adeno CA

All the patients were started on LA therapy at the time of diagnosis. Four patients progressed on LA therapy, and two patients died on LA therapy within 3 months. Among those four patients who progressed on LA therapy, three received chemotherapy on progression with liver metastasis. As the duration of response was short to docetaxel, two of three patients received Ab after progression on chemotherapy and died while taking the Ab. The third patient died while on chemotherapy. The fourth patient is alive with stable disease (enzalutamide was started after LA therapy) (Table 3).

**Table 3:** Outcome of Patients Treated With Hormone

Treatment characteristics	No. of patients	Response
Lupron + casodex who presented with liver metastasis	2/2	Expired in less than 3 months
Zytiga (Ab) after taxotere	2/3	Progression
Progress on LA then on xtandi (enzalutamide)	1/6	Stable disease

Chemotherapy of choice was docetaxel (75 mg/m^2^, every 3 weeks with prednisone) in three patients, as mentioned above. Median number of cycles given was 4 (range 3 - 6 cycles). A PSA drop of > 50% was seen in two patients while one had a > 90% decrease on docetaxel. Median survival times as calculated from the time of diagnosis of prostate cancer to death and from development of liver metastasis to death were 3.25 years and 4 months, respectively; none had treatment-related deaths.

## Discussion

Prostate cancer has a well-known predilection for bony metastasis. In a single institution retrospective analysis of 10 years, 620 cases of prostate cancer were reviewed, out of which only 30 cases of liver metastasis were found (4.7%) [6]. In another recent series (1,045 patients) presented as an abstract at the 2012 ASCO annual meeting by Kelly et al, the incidence of hepatic metastasis was only 5.6% [7]. Therefore, hepatic metastasis represents a rare occurrence. Neuroendocrine differentiation is often seen in prostatic adenocarcinoma and is hypothesized to result from long-term androgen deprivation therapy [8, 9]. To rule out liver metastasis of neuroendocrine origin in our patients, liver biopsy specimens of five patients were examined and found to be negative for neuroendocrine markers.

Median overall survival in our patients after development of liver metastasis was only 4 months. Another series of 28 patients by Pouessel et al showed a median overall survival of 6 months only after development of liver metastasis [10]. In a much larger series of 1,045 patients presented by Kelly et al [7], median survival of 14.4 months was reported in patients with liver metastasis compared to 22.2 months without liver metastasis. Therefore, development of liver metastasis in patients with prostate adenocarcinoma represents an ominous prognosis.

To date, no literature is available specifically regarding hormonal therapy or chemotherapy in the treatment of liver metastasis secondary to prostate carcinoma. In our small series of six patients (Table 3), we found that two patients who presented with liver metastasis deteriorated rapidly despite LA therapy and died in 2.5 months and 3 months, respectively, prior to the initiation of any other form of therapy; of three patients who developed liver metastasis early in the course of LA therapy, two were treated with Ab and progressed. Our fifth patient progressed to liver metastasis within 6 months of LA therapy and recently started enzalutamide. He continues to be monitored closely. All of our six patients showed no response to hormonal therapy, i.e., LA or Ab. This observation suggests that hormonal manipulation with either LAs or Ab therapy is not effective in the setting of liver metastasis. The response to androgen receptor antagonist therapy in such a setting is currently unknown. It will be interesting to see the long-term response of our chemonaive patient on enzalutamide.

In our series, three out of six patients who received chemotherapy showed good response; two of these patients showed a PSA decrease of more than 50% while the third patient had more than 90% decrease. However, these responses were of short duration (Table 4). In Kelly et al series, 55% of patients with liver metastasis responded to docetaxel chemotherapy compared with 64% who did not have liver metastasis [7]. In another series of 14 patients presented at a 2011 symposium in abstract form, an alternate regimen of epirubicin, cisplatin, and fluorouracil was used in patients with liver metastasis. Although a higher response rate was achieved, it was of short duration [11]. No literature addresses the response to hormone treatment in hepatic metastasis in prostate carcinoma including two large series of reports [7, 12]. We speculate here that liver metastasis in prostate carcinoma does not respond to hormonal manipulation therapy (LAs or Ab). It does respond to cytotoxic chemotherapy, but the responses are brief. A larger series of patients is needed to substantiate our speculation.

**Table 4:** Treatment With Chemotherapy

Treatment characteristics: chemotherapy	Number of patients
Chemotherapy after liver metastasis (taxotere)	3 (four cycles ranging from three to six cycles)
PSA drop after taxotere	> 50% (2), > 90% (1)
Total survival from diagnosis of prostate cancer	3.25 years
Survival after liver metastasis	4 months

Approximately 67% of patients with prostate carcinoma are initially responsive to androgen deprivation therapy [13, 14]. One-third of treated prostate cancer patients experience recurrence and will progress into castrate-resistant prostate cancer (CRPC) [15]. Numerous studies have shown that androgen receptor (AR)-mediated signaling plays an important role in the development of CRPC, which may render prostate cancer cells resistant to treatment [16, 17]. The possible mechanisms are AR overexpression, AR amplification, or AR mutation. These mechanisms are hypothesized to be the driving force behind progression of prostate cancer even in the presence of minimal androgen levels [18, 19]. An alternative mechanism of AR activation has also been described as cross-talk with other cellular signaling pathways leading to tumor growth and CRPC progression [20-23]. The latest finding regarding the AR-V7 mutation, which is resistant to enzalutamide and Ab, is the prime example [24]. Since liver metastasis in prostate cancer represents an aggressive subset, these AR mutations, overexpression, amplification, and cross-talk mechanisms may explain the refractory nature of such patients to hormonal agents. A detailed study of AR mutation, amplification, and other cross-talk pathways will be necessary to answer these questions.

Our patients’ characteristics of high Gleason score (Table 1) and absence of PSA staining in the liver in most of our patients is indicative of high-grade malignancy [25]. Whether there is a correlation between such high-risk characteristics and liver metastasis cannot be determined from our small study. Any future validation of this hypothesis will need to involve more cases.

### Conclusion

Liver metastasis in patients with mCRPC represents a subset with poor outcome. Scant data are available on managing such patients. Our single-institution retrospective study of six patients with liver metastasis suggests that such patients represent an aggressive subset of prostate cancer patients who do not respond to hormonal therapy (LA and Ab). Although they may respond biochemically to docetaxel, the responses are of short duration. A larger, multicenter data set should be collected and reviewed to better characterize this aggressive subset of mCRPC with an aim to explore and identify an alternative therapeutic regimen for these patients.
